# Supplementary material for: Content-rich biological network constructed by mining PubMed abstracts
Source: BMC Bioinformatics. 2004 Oct 8;5:147. doi: 10.1186/1471-2105-5-147 (PMC528731; doi:10.1186/1471-2105-5-147)
Supplement: Additional File 2 — The original results of the above study (non-essential files are deleted to keep the file size under the limit set by BMC bioinformatics). [file 1471-2105-5-147-S2.bz2 › chilibotAdditionalFile2/dip10/45ID7597030E179/html/TAF9_GTF2B.html]

 


 **TAF9** and **GTF2B** 
  
Found 5 abstracts in PubMed, retrieved 5.  
 

 What does Google say? 
 PDF only 
| .edu only 

---

**Interactive relationship** (e.g. stimulation, inhibition, etc)

**Neutral relationship**- Interestingly, we observed that N CoR expression ablated the functional interaction between TFIIB  [ **GTF2B** ]  and TAFII32  [ **TAF9** ]  that is critical to the initiation of transcription.  Ref: 9611234 Nucleic Acids Res, 1998

**Non-interactive relationship** (e.g. studied together, co-existance, homology, etc.)

- The corepressor N CoR and its variants RIP13a and RIP13Delta1 directly interact with the basal transcription factors TFIIB  [ **GTF2B** ] , TAFII32  [ **TAF9** ]  and TAFII70.  Ref: 9611234 Nucleic Acids Res, 1998
- In this study, 14 3 3 proteins are shown to bind the TATA box binding protein TBP, transcription factor IIB  [ **GTF2B** ]  TFIIB  [ **GTF2B** ] , and the human TBP associated factor  [ **TAF9** ]  hTAF II 32 in vitro but not hTAF II 55.  Ref: 10449590 Plant Cell, 1999
